# Supplementary material for: PDPN Is Expressed in Various Types of Canine Tumors and Its Silencing Induces Apoptosis and Cell Cycle Arrest in Canine Malignant Melanoma
Source: Cells. 2020 May 5;9(5):1136. doi: 10.3390/cells9051136 (PMC7290317; doi:10.3390/cells9051136)
Supplement: Supplementary file 1 [file cells-09-01136-s001.zip › Supplementary Table/Table S1.docx]

Table S1 The list of antibodies.

| Antibodies | Application and condition |
| --- | --- |
| dPDPN (Pmab-48, ZENOAQ Resource) | IHC^5^, 50 µg/ml in TBS-t with 10% skim milk  WB^6^, dilution 1:100 in TBSt-milk |
| Ki67 (Dako^1^, #M7240) | IHC^5^, Ready to use |
| dPDPN (Pmab-38, ZENOAQ Resource) | FACS, dilution 1:500 |
| Phospho-ATM (Ser1981) (Merck Millipore, #05-740) | WB^6^, dilution 1:1000 in TBSt-milk |
| Phospho-cdc2 (Thr15) (CST^2^, #4539) | WB^6^, dilution 1:1000 in TBSt-BSA |
| Total-cdc2 (CST^2^, #77055S) | WB^6^, dilution 1:1000 in TBSt-BSA |
| Phospho-chk1 (Ser345) (CST^2^, #2348) | WB^6^, dilution 1:1000 in TBSt-BSA |
| Phospho-chk2 (Thr68) (CST^2^, #2197) | WB^6^, dilution 1:1000 in TBSt-BSA |
| Phospho-p53 (Ser15) (CST^2^, #9286) | WB^6^, dilution 1:500 in TBSt-BSA |
| Total-p53 (BD, #610183) | WB^6^, dilution 1:500 in TBSt-BSA |
| Cyclin B1 (BD, #554177) | WB^6^, dilution 1:1000 in TBSt-BSA |
| p21 (Santa Cruz Biotechnology^3^, #sc-397) | WB^6^, dilution 1:500 in TBSt-milk |
| Cleaved Caspase 3 (CST^2^, #9661) | WB^6^, dilution 1:500 in TBSt-BSA |
| Caspase 3 (CST^2^, #9662) | WB^6^, dilution 1:500 in TBSt-BSA |
| Actin (Merck Millipore^4^, #MAB1501) | WB^6^, dilution 1:10000 in TBSt-milk |

^1^ Dako Japan, Kyoko, Japan; ^2^ Cell Signaling Technology, danvers, MA, USA;

^3^ Santa Cruz Biotechnology, Dallas, TX, USA; ^4^ Merck Millipore, Burlington, MA, USA;

^5^ Immunohistochemistry; ^6^ Western blotting.
