# Supplementary material for: PDPN Is Expressed in Various Types of Canine Tumors and Its Silencing Induces Apoptosis and Cell Cycle Arrest in Canine Malignant Melanoma
Source: Cells. 2020 May 5;9(5):1136. doi: 10.3390/cells9051136 (PMC7290317; doi:10.3390/cells9051136)
Supplement: Supplementary file 1 [file cells-09-01136-s001.zip › Supplementary Table/Table S2.docx]

Table S2 The list of cell lines.

| Tissue of origin | Cell line | References |
| --- | --- | --- |
| Canine transitional cell carcinoma | LOVE^a^ | [80,81] |
|  | SORA^a^ | [80,81] |
|  | TCCUB^a^ | [80,81] |
| Canine mammary carcinoma | CHMp^a^ | [82] |
|  | CIPp^a^ | [82] |
|  | CIPm^a^ | [82] |
| Canine osteosarcoma | OOS^a^ | [83] |
|  | POS^a^ | [84] |
|  | HMPOS^a^ | [84] |
| Canine malignant melanoma | CMM11^b^ | [85] |
|  | Mi^a^ | [86] |
|  | CMM12^b^ | [78] |
|  | CMM9^b^ | [87] |
|  | CMM10^b^ | [87] |
|  | KMeC^a^ | [88] |
|  | LMeC^a^ | [88] |
|  | Pu^a^ | [86] |
| Chinese hamster ovary | CHO/dPDPN^b,c^ | [24] |
|  | CHO^b,c^ | [24] |

^a^ Cultured in RPMI1640 supplemented with gentamicin and 10% FBS.

^b^ Cultured in DMEM/Ham's F-12 supplemented with penicillin, streptomycin, and 10% FBS

^c^ CHO: Chinese hamster ovary
